# Pilot study: Effects of ovariectomy-induced estrogen deficiency on the biomechanical and structural properties of the intact anterior cruciate ligament in a porcine model

**DOI:** 10.1051/sicotj/2026017

**Published:** 2026-04-29

**Authors:** Hibiki Kakiage, Tsuneari Takahashi, Yuji Kaneda, Wataru Kurashina, Katsushi Takeshita, Hirotaka Chikuda

**Affiliations:** 1 Department of Orthopaedic Surgery, Gunma University Maebashi Japan; 2 Department of Orthopaedics, Jichi Medical University Shimotsuke Japan; 3 Medical Simulation Center, Department of Surgery, Division of Gastroenterological, General and Transplant Surgery, Jichi Medical University Shimotsuke Japan; 4 Rehabilitation Center Tochigi Medical Center Shimotsuga Tochigi Japan

**Keywords:** Anterior cruciate ligament, Ovariectomy, Estrogen deficiency, Biomechanics, Porcine model

## Abstract

*Introduction*: This pilot study investigated the effects of ovariectomy-induced estrogen deficiency on the biomechanical properties of intact anterior cruciate ligaments (ACLs) in a porcine model, a biological condition that may influence ligament integrity and injury susceptibility. *Methods*: A bilateral ovariectomy model was used to induce systemic estrogen deficiency. Fourteen two-month-old female pigs were included. Four pigs (8 knees) underwent bilateral ovariectomy (OV group). The left knees of 10 pigs that underwent laparotomy without ovariectomy for a separate study were analyzed as controls (C group). At 12 weeks, knee joints were examined macroscopically, followed by biomechanical testing consisting of cyclic anterior drawer loading and load-to-failure. *Results:* All ACLs were intact without arthrofibrosis or cartilage degeneration. During cyclic testing, anterior tibial translation was significantly lower in the OV group compared with controls (0.47 ± 0.14 mm vs. 0.82 ± 0.32 mm, *P* = 0.017). Failure mode differed between groups: all posterolateral bundles in controls avulsed at their insertions, whereas six of eight in the OV group ruptured in the midsubstance (*P* = 0.0070). No significant between-group differences were observed in yield load, maximum load, stiffness, or elongation at failure. *Conclusion*: Ovariectomy-induced estrogen deficiency altered ACL failure characteristics and reduced translation without affecting ultimate strength. These findings suggest that ovarian hormone deficiency compromises ligament quality, providing a potential mechanism for increased ACL injury risk in young female athletes. These findings should be interpreted as pilot, hypothesis-generating data. *Level of Evidence*: Experimental laboratory study.

## Introduction

The anterior cruciate ligament (ACL) is among the knee ligaments most prone to injury, with female athletes experiencing a markedly higher risk – estimated at two- to ten-fold greater – compared with their male counterparts. Previous translational research using large-animal models demonstrated sex-based differences, showing that female pigs exhibited inferior graft strength, greater joint laxity, and more pronounced cartilage damage after a 15-week healing period [1]. In addition to these sex-specific disparities, disturbances in the menstrual cycle, which are frequently observed in female athletes, have been recognized as a contributing factor to musculoskeletal vulnerability [2]. Secondary functional hypothalamic amenorrhea (SFHA) is common among female athletes, especially in weight-sensitive sports [3]. Estrogen deficiency has been recognized as a potential biological factor affecting musculoskeletal tissues, including ligaments.

Experimental evidence has also indicated that estrogen deficiency following ovariectomy leads to thinning of femoro-tibial cartilage and alterations in both biomechanical and histological parameters, implicating disorganization of proteoglycan and collagen networks as an underlying mechanism. Such alterations are accompanied by increased expression of inducible nitric oxide synthase (iNOS), which may accelerate matrix degradation [4]. Since excessive iNOS activation after injury has been linked to enhanced apoptosis of ACL fibroblasts, impaired self-repair of the ligament may result.

Despite these findings, limited data are available regarding how ovarian hormone deprivation affects the intrinsic properties of intact ACL tissue. We therefore postulated that ovariectomy-induced estrogen deficiency compromises the biomechanical properties and structural organization of the native ACL in a porcine model [5]. The aim of the present pilot investigation was to test this hypothesis.

## Methods

### Animal experiments

All animal procedures were approved by the Animal Care and Use Committee of the Institute of the corresponding author (Approval no. 24107-01, October 10, 2024) and were conducted in compliance with institutional guidelines. A total of 14 two-month-old female pigs (Sanesu Breeding, Funabashi, Japan; weight range, 24.0–32.0 kg) were included. In this breed, regular estrous cycles are typically established after the onset of puberty at approximately 5–6 months of age. Therefore, animals in the present study were pre-pubertal, allowing controlled induction of estrogen deficiency via ovariectomy without confounding cyclic hormonal fluctuations. Four pigs (8 knees) were assigned to the ovariectomy group (Group OV). The remaining 10 pigs underwent ovariectomy-free laparotomy for a separate ligament reconstruction model in which the right hindlimb was used; in the present study, however, only the contralateral left hindlimbs were analyzed and served as controls (Group C).

### Surgical procedures in group OV

After overnight fasting, pigs were premedicated with intramuscular medetomidine (0.06 mg/kg), midazolam (0.3 mg/kg), and atropine (0.02 mg/kg). General anesthesia was induced and maintained with endotracheal intubation and sevoflurane. All operations were performed under aseptic conditions by an experienced gastroenterological and general surgeon (Y.K.). For infection prophylaxis, cefazolin sodium hydrate (1 g) was administered intravenously intraoperatively. A 10-cm lower midline laparotomy was performed. After peritoneal incision, the pelvic cavity was exposed. The uterus, both ovaries, and fallopian tubes were identified. The ovarian arteries and veins, together with surrounding tissues, were carefully ligated with 2-0 silk sutures, and the ovaries were excised. The peritoneal cavity was irrigated with saline, and meticulous hemostasis was confirmed. To reduce postoperative adhesions, a Seprafilm adhesion barrier (Baxter, IN, USA) was applied to the intestinal serosa. The abdominal wall was then closed in layers: peritoneum with running 2-0 Vicryl (ETHICON, IN, USA), fascia with interrupted 1-0 Vicryl, and skin with subcuticular 4-0 PDS II (ETHICON, IN, USA). For postoperative analgesia, a 16.8 mg fentanyl patch was applied for 72 h.

### Postoperative management

**Figure 1:**
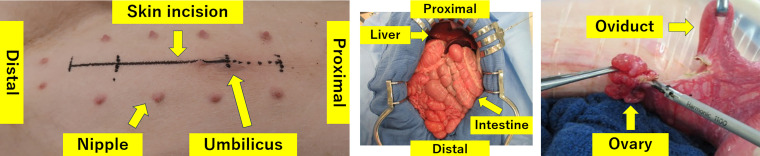
Surgical procedure of bilateral ovariectomy in the porcine model. (A) Skin marking of a 10-cm lower midline incision. (B) Peritoneal exposure through the incision. (C) Complete excision of the ovary.

Animals were returned to their cages (2 × 3 × 2 m) immediately after surgery and allowed unrestricted weight-bearing. Clinical monitoring was performed one to two times per week to evaluate wound healing, gait, and the presence of limping or discharge. All pigs recovered normal gait within 2 days after surgery. The effects of ovariectomy-induced estrogen deficiency were assessed at 12 weeks postoperatively, in line with previous evidence on the timing of structural changes. At euthanasia, body weight ranged from 44.3 to 51.6 kg. A repeat 10-cm lower midline laparotomy confirmed the absence of infection, adhesions, or incomplete resection. Both knee joints were grossly examined for secondary changes, including synovitis, cartilage degeneration, ACL alterations, and meniscal lesions. Specimens were harvested immediately thereafter. The femur and tibia were transected 13 cm from the joint line, and all surrounding soft tissues – including muscles, the patella, patellar tendon, and non-ACL ligaments – were excised to avoid meniscal damage. The fibula was cut distal to the lateral collateral ligament attachment. Both the femur and tibia were then potted separately into aluminum tubes using bone cement (Figure 1). All animals were euthanized at 12 weeks postoperatively, according to the Animal Care and Use Committee regulations. Euthanasia was performed via intravenous administration of potassium chloride following induction with general anesthesia to ensure loss of consciousness.

### Biomechanical evaluations

#### Anterior drawer testing

**Figure 2:**
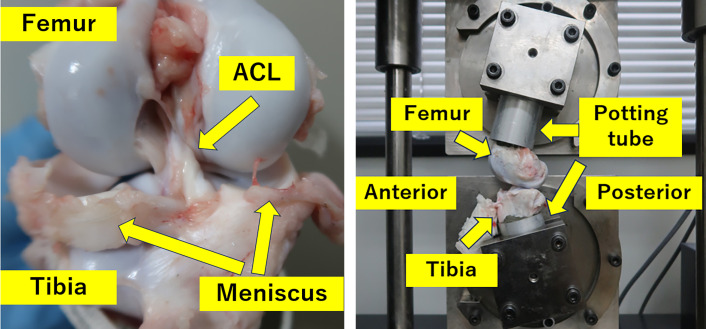
Preparation and fixation of the femur-ACL-tibia complex for biomechanical testing. (A) The knee joint was exposed after euthanasia, and all soft tissues except the ACL carefully removed. (B) The femur and tibia were transected 13 cm from the joint line and embedded into aluminum tubes using bone cement to ensure rigid fixation during biomechanical testing.

Specimens were kept moist with saline spray during testing. Axial translation was measured under a standardized drawer force as previously reported. Knee specimens were mounted on a tensile tester (Tensilon RTG-1250; Orientec, Tokyo, Japan) with custom grips, positioning the tibia at 45° of flexion relative to the femur (Figure 2). Preconditioning included a static preload of 5 N for 30 s, followed by 20 cycles of loading between 0 and 40 N at a cross-head speed of 100 mm/min. Axial translation after the 20th cycle was recorded using Tensilon Advanced Controller software (Orientec, Tokyo, Japan).

#### Structural testing of the femur–ACL–tibia (FAT) complex

After cyclic testing, the menisci were carefully excised while preserving the native ACL. The FAT complex was mounted on the tensile tester, with the tibia fixed at 45° flexion relative to the femur to apply tensile load along the ACL axis (Figure 2). Preconditioning consisted of a 5 N static preload for 10 min and 10 cycles of loading/unloading (3% strain) at 20 mm/min. Specimens were then loaded to failure at 50 mm/min. Failure modes were documented, and load–elongation curves were generated. Structural parameters, including yield load, maximum load, linear stiffness, and elongation at failure, were calculated using the software. Three-plane alignment was carefully verified during mounting to ensure colinear loading along the ACL axis and to minimize off-axis forces.

Preconditioning at 5 N was intentionally set below the viscoelastic threshold to standardize specimen seating while minimizing pre-damage prior to cyclic testing, consistent with previously reported porcine knee drawer-testing protocols.

### Statistical analysis

Each knee was analyzed as an independent specimen, and no contralateral pairing was performed. Control knees were derived from different animals (left hindlimbs only). This design was chosen to avoid within-animal dependency in biomechanical testing.

Continuous data were expressed as means ± standard deviations and compared using Student’s *t*-tests. Ordinal data were reported as medians (range) and compared with Mann–Whitney *U* tests, while categorical variables were analyzed with Fisher’s exact test. A priori power analysis was based on anterior tibial translation values reported by Takahashi et al. in a large-animal ACL reconstruction model, indicating that eight specimens per group would provide 80% power to detect group differences (*α* < 0.05). All analyses were conducted with EZR software, with *p* < 0.05 considered statistically significant.

## Results

### Gross observations in the knee joint

After the surgery, no signs of arthrofibrosis were observed. All ACLs were intact and covered with synovial tissues. No obvious degenerative changes on the articular cartilage were observed at the time of euthanasia.

### Biomechanical evaluations

#### Drawer testing

The anterior tibial translation during the cyclic testing was 0.47 ± 0.14 mm in Group OV and 0.82 ± 0.32 mm in Group C, with a significant difference observed between the two groups (*P* = 0.017) (Table 1).

**Table 1:** Anterior tibial translation during cyclic loading.

	C group (*n* = 10)	OV group (*n* = 8)	*p* value**
Anterior tibial translation*	0.82 ± 0.32	0.47 ± 0.14	0.017

*Data are expressed as mean ± standard deviation unless otherwise indicated.

**Student’s *t*-test.

#### Observation of failure mode on tensile testing

**Figure 3:**
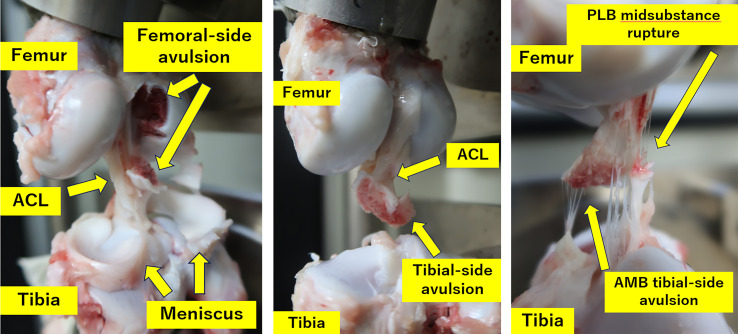
Representative failure modes of the ACL. (A) Femoral-side avulsion in the control group. (B) Tibial-side avulsion in the control group. (C) Midsubstance rupture in the ovariectomy group. (A, B) Femoral- and tibial-side avulsions commonly observed in controls. (C) Midsubstance rupture frequently observed in the ovariectomy group.

During tensile testing, all ACLs in Group C had avulsed from the femoral or tibial attachment for both the anteromedial bundle (AMB) and the posterolateral bundle (PLB). On the other hand, even though all AMBs in Group OV had avulsed from the femoral or tibial attachment, six out of eight PLBs in Group OV had ruptured in the midsubstance of the bundle (*P* = 0.0070) (Table 2, Figure 3).

**Table 2:** Failure modes of the ACL under tensile loading.

	C group (*n* = 10)	OV group (*n* = 8)	*p* value*
Avulsion	10 (100%)	2 (25%)	
Midsubstance rupture	0 (0%)	6 (75%)	0.007

*Fisher’s exact test.

In the ovariectomy group, midsubstance rupture occurred exclusively in the posterolateral bundle, whereas all anteromedial bundle failures were avulsions in both groups.

#### Structural properties of the FAT complex

No significant differences were observed in the upper yield load (Group OV, 662.4 ± 328.1 N; Group C, 649.7 ± 270.7 N, *P* = 0.93), maximum load (Group OV, 911.1 ± 218.7 N; Group C, 846.1 ± 221.6 N, *P* = 0.54), linear stiffness (Group OV, 109.2 ± 43.1 N/mm; Group C, 84.9 ± 43.0 N/mm, *P* = 0.25), and elongation at failure (Group OV, 10.4 ± 3.2 mm; Group C, 13.4 ± 8.6 mm, *P* = 0.37) between groups (Table 3).

**Table 3:** Structural properties of the femur-ACL-tibia complex under load-to-failure testing.

Parameter	C group (*n* = 10)	OV group (*n* = 8)	*p* value**
Yield load (N)*	649.7 ± 270.7	662.4 ± 328.1	0.93
Maximum load (N)*	846.1 ± 221.6	911.1 ± 218.7	0.54
Linear stiffness (N/mm)*	84.9 ± 43.0	109.2 ± 43.1	0.25
Elongation at failure (mm)*	13.4 ± 8.6	10.4 ± 3.2	0.37

*Data are expressed as mean ± standard deviation unless otherwise indicated.

**Student’s *t*-test.

## Discussion

The present study demonstrated that ovariectomy-induced estrogen deficiency altered the functional and biomechanical behavior of the intact ACL in a porcine model. Specifically, cyclic anterior tibial translation was significantly reduced, which may reflect altered viscoelastic behavior of the ligament, and the failure pattern shifted toward midsubstance rupture, particularly in the posterolateral bundle. These findings indicate that estrogen deficiency affects ligament viscoelasticity and collagen organization, potentially compromising functional resilience without reducing ultimate strength.

Estrogen regulates ligament metabolism through ER-α and ER-β signaling, influencing collagen turnover, matrix metalloproteinase (MMP) activity, and nitric oxide production [4–7]. Estrogen deficiency reduces type I collagen expression and increases matrix-degrading enzyme activity, leading to fibrillar disorganization and loss of viscoelasticity [4, 8–10]. These mechanisms likely explain the observed combination of increased stiffness and altered failure mode under estrogen-deficient conditions. Such cellular and structural changes parallel those reported in aging or postmenopausal connective tissues [8, 11–13]

Clinically, these findings provide biomechanical evidence that hypoestrogenic conditions observed in certain athletic or metabolic states may impair ligament resilience and alter failure characteristics, predisposing to non-contact ACL injury [8, 14]. Monitoring menstrual health and considering hormonal or training-based interventions could represent important preventive strategies [1–3].

Bilateral ovariectomy induces an abrupt hypoestrogenic state and does not replicate the gradual, reversible endocrine changes observed in functional hypothalamic amenorrhea in adolescent athletes. Accordingly, the present model should be interpreted as representing estrogen deficiency per se, providing a mechanistic boundary condition rather than a direct simulation of clinical FHA [15]. These findings should therefore be interpreted as reflecting the effects of estrogen deprivation rather than a direct experimental model of functional hypothalamic amenorrhea. Future studies incorporating histological or ultrastructural analyses, such as collagen fiber orientation or extracellular matrix composition, may help clarify the structural mechanisms underlying the biomechanical observations reported in the present pilot study.

This study has several limitations that should be acknowledged. First, the design of the control group represents a limitation. Control knees were obtained from animals that underwent laparotomy for a separate ligament reconstruction experiment, and only the contralateral limb was used in the present analysis. Although this approach allowed efficient use of experimental animals in accordance with ethical guidelines, systemic effects of surgery or potential laterality bias cannot be completely excluded. Therefore, subtle biomechanical differences between groups should be interpreted with caution. Because only the contralateral limb of the animals in the control experiment was analyzed, potential laterality-related biomechanical bias cannot be completely excluded. Second, the evaluation was limited to 12 weeks postoperatively, and the long-term effects of ovariectomy-induced estrogen deficiency on ligament properties were not assessed. Third, the number of animals in each group was relatively small, which may have limited the statistical power to detect subtle differences. Fourth, serum estrogen level was not measured. Finally, although the procedures were carried out by an experienced surgeon, variability related to surgical technique cannot be completely excluded. Despite these limitations, this study provides important pilot data on the biomechanical and histological impact of ovarian hormone deficiency on the intact ACL. The present study provides foundational data that may guide future clinical research aimed at addressing sex-specific considerations in ACL injury prevention and reconstruction.

## Conclusions

Ovariectomy-induced estrogen deficiency led to altered biomechanical behavior of intact ACLs in a porcine model, with reduced translation during cyclic loading and a shift in failure mode toward midsubstance rupture. Although no differences were found in ultimate structural strength, these results suggest that ovarian hormone deficiency may compromise ligament quality and provide a potential biological explanation for the higher ACL injury risk observed in young female athletes. This pilot study offers foundational data for future translational and clinical investigations into sex-specific strategies for ACL injury prevention and management.

**Abbreviations:**
ACL: Anterior Cruciate Ligament
iNOS: Inducible Nitric Oxide Synthase
OV group: Ovariectomy Group
C group: Control Group
FAT: Femur–ACL–Tibia
AMB: Anteromedial Bundle
PLB: Posterolateral Bundle
MMP: Matrix Metalloproteinase

## Data Availability

The datasets generated and analyzed during the current study are available from the corresponding authors upon reasonable request.
